# Comorbidity in chronic kidney disease: a large cross-sectional study of prevalence in Scottish primary care

**DOI:** 10.3399/bjgp20X714125

**Published:** 2021-02-09

**Authors:** Clare MacRae, Stewart W Mercer, Bruce Guthrie, David Henderson

**Affiliations:** Usher Institute of Population Health Sciences and Informatics, University of Edinburgh, Edinburgh.; Usher Institute of Population Health Sciences and Informatics, University of Edinburgh, Edinburgh.; College of Medicine and Veterinary Medicine, University of Edinburgh, Edinburgh.; Usher Institute of Population Health Sciences and Informatics, University of Edinburgh, Edinburgh.

**Keywords:** chronic kidney disease, comorbidity, epidemiology, general practice, renal insufficiency, chronic

## Abstract

**Background:**

Chronic kidney disease (CKD) is commonly comorbid with hypertension, diabetes, and cardiovascular disease (CVD). However, the extent of comorbidity in CKD across a range of concordant (shared pathophysiology and/or treatment) conditions and discordant (unrelated pathophysiology and/or different or contradictory treatment) conditions is not well documented.

**Aim:**

To ascertain the prevalence of comorbidity, across 39 physical and mental health comorbidities, in adults with CKD in a large, nationally representative primary care population.

**Design and setting:**

Cross-sectional analysis of a primary care dataset representing 1 274 374 adults in Scotland.

**Method:**

This study was a secondary analysis of general practice electronic medical record data using binary logistic regression models adjusted for age, sex, and socioeconomic status. Data of adults aged ≥25 years and 40 long-term conditions were used.

**Results:**

A total of 98.2% of adults with CKD had at least one comorbidity, versus 51.8% in controls. After adjustment for age, sex, and deprivation, people with CKD were more likely to have 1 (adjusted odds ratio [aOR] 6.5, 95% confidence interval [CI] = 6.0 to 7.1), 2–3 (aOR 15.2, 95% CI = 14.0 to 16.5), 4–6 (odds ratio [OR] 26.6, 95% CI = 24.4 to 28.9), and ≥7 other conditions (OR 41.9, 95% CI = 38.3 to 45.8). Furthermore, all concordant (seven out of seven), the majority of discordant physical health conditions (17 out of 24), and mental health conditions (six out of eight) had statistically significant positive associations with CKD after adjustment.

**Conclusion:**

Chronic kidney disease is associated with extreme comorbidity across a wide range of mental and physical conditions. Routine care for people with CKD should include recognition and management of comorbidities, and clinical guidelines should support clinicians to do this.

## INTRODUCTION

Chronic kidney disease (CKD) is a leading cause of mortality and morbidity,^1^^,^^2^ and commonly occurs in people with coexistent comorbidity,^3^^,^^4^ which is associated with adverse clinical outcomes.^5^ Risk of death in people with CKD rises exponentially as kidney function deteriorates, largely attributable to cardiovascular disease (CVD).^6^^,^^7^ Diabetes and hypertension are the leading causes of CKD^6^^,^^8^ and are risk factors for the progression of both CKD and CVD. Targeting modifiable risk factors can therefore improve survival and quality of life by reducing CVD in those with CKD,^9^ and progression of CKD to end-stage renal dysfunction (ESRD).^1^^,^^7^^–^^9^

CKD is associated with complications that affect all body systems,^1^ and people with CKD experience significantly lower health-related quality of life compared with the general population.^6^ The coexistence of CKD in the context of both concordant comorbidities (those with shared pathophysiology and/or pharmacological treatments) and non-concordant conditions (where pathophysiology is unrelated and/or where treatments of different conditions are complicating or contradictory) is associated with increased healthcare utilisation, length of inpatient hospital stay, and mortality.^3^^,^^10^^,^^11^ Comorbidity and polypharmacy are common in CKD, even in its early stages, and are associated with use of potentially hazardous nephrotoxic medications^12^ and significant treatment burden affecting a person’s ability to cope with treatment.^4^^,^^13^

There is a marked social gradient in the effects of CKD where low socioeconomic status (SES) is associated with worse CVD and mortality outcomes.^14^^,^^15^ People of lower SES are over-represented in those who develop CKD.^16^ Though CKD is known to be associated with concordant comorbidity, in particular potentially causal diseases such as hypertension and diabetes,^11^ the majority of the existing literature examines a limited number of comorbid conditions and without reference to SES.^3^^,^^11^ Thus, there is a gap in evidence regarding the broader range of conditions that are typically managed in primary care, which include concordant and discordant physical conditions and mental health conditions. This study examines prevalence of a wide range of comorbid conditions in people with and without CKD, using nationally representative primary care data from a large dataset in Scotland.^17^

## METHOD

This study used data obtained from the Primary Care Clinical Informatics Unit at the University of Aberdeen of 1 274 374 adults aged ≥25 years, who were alive and permanently registered with 314 general practices on the 31 March 2007; 31% of all practices in Scotland. These practices had recorded routine electronic clinical data as part of the Scottish Programme for Improving Clinical Effectiveness in Primary Care (SPICE-PC), a voluntary scheme run by the Scottish Government. The dataset is a nationally representative sample in terms of patients’ age, sex, and SES,^18^ and was created for a previous study examining multimorbidity.^17^

**Table:** How this fits in

Chronic kidney disease (CKD) is common and results in significant mortality and morbidity, and is known to be commonly associated with hypertension, diabetes, and cardiovascular disease. Despite research indicating that people with CKD and comorbidity, of any type, are at increased risk of adverse clinical outcomes, little is known about the prevalence of discordant physical and mental health conditions in people with CKD. The present study found that almost all people with CKD have coexisting comorbidities, and that extreme comorbidity is >40 times more common in adults with CKD compared with age-, sex-, and deprivation-adjusted controls. The majority of discordant physical and mental health conditions were more common in people with CKD.

SES was measured using the Carstairs index,^19^ which assigns SES based on postcode of residence and was grouped into deciles (equal tenths of the population ranked by Carstairs Index). This score uses four indicators judged to represent material disadvantage in the population taken from census data (lack of car ownership, low occupational social class, overcrowded households, and male unemployment).

The dataset contained information on age, sex, SES, and 40 long-term conditions, made up of 32 physical health (including CKD) and eight mental health conditions. The physical health conditions categorised as concordant with CKD were hypertension, peripheral vascular disease (PVD), heart failure, stroke/transient ischaemic attack (TIA), atrial fibrillation (AF),^11^ diabetes, and coronary heart disease (CHD),^3^ according to classification from previous studies. Remaining physical health conditions and mental health conditions were classified as discordant (Box 1).

**Box 1.** Classification of diseases according to type in association with CKD

**Concordant physical health condition**	**Discordant physical health condition**	**Mental health condition**
Hypertension	Rheumatological conditions	Depression
Peripheral vascular disease (PVD)	Inflammatory bowel disease (IBD)	Anxiety and other neurotic stress-related and somatoform disorders
Heart failure	Painful condition	Alcohol problems
Stroke and transient ischaemic attack	Thyroid disorders	Other psychoactive substance misuse
Atrial fibrillation (AF)	Chronic obstructive pulmonary disease	Schizophrenia or bipolar affective disorder
Diabetes	Bronchiectasis	Dementia
Coronary heart disease (CHD)	Chronic sinusitis	Learning disability
	Migraine	Anorexia or bulimia
	Diverticular disease of intestine	
	Viral hepatitis	
	Irritable bowel disease	
	Constipation	
	Psoriasis or eczema	
	Prostate disorders	
	Epilepsy	
	Hearing loss	
	Glaucoma	
	Chronic liver disease	
	Blindness and low vision	
	New diagnosis of cancer in last 5 years	
	Parkinson’s disease (PD)	
	Multiple sclerosis	
	Dyspepsia	
	Asthma	

CKD = chronic kidney disease.

Individuals were identified as having CKD if their primary care electronic medical record (EMR) contained a code for CKD as part of the CKD disease register for the Quality and Outcomes Framework (QOF — the UK national pay-for-performance system that incentivised recording and care for CKD stages 3 to 5). No individuals in this analysis had missing data for variables and therefore none were excluded from the study. The control group was defined as the entire population aged ≥25 years without a code for CKD in their EMR.

The overall number of physical and mental health conditions for each individual was documented. Binary logistic regression models were used, with presence of CKD as the outcome variable and report odds ratios (ORs) with 95% confidence intervals (CIs) for explanatory variables. Unadjusted and adjusted models for age, sex, and SES were calculated. A *P*-value of <0.05 was considered statistically significant. All statistical analyses were performed in R (version 4.0.2).

## RESULTS

### Participant demographics

**Figure 1.**
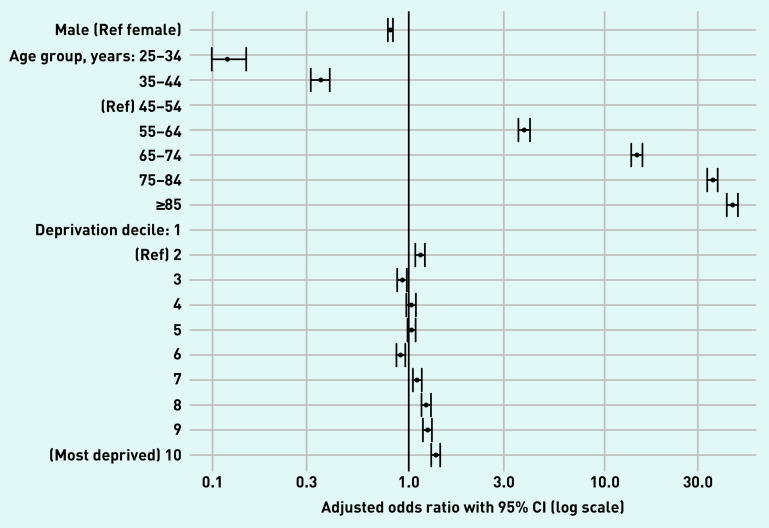
***Age, sex, and deprivation-adjusted odds ratios for demographics by chronic kidney disease status. CKD group N = 33 567, non-CKD group N = 1 240 807. CKD = chronic kidney disease.***

A total of 1 274 374 individuals were included in the study, of which 33 567 (prevalence = 2.6%) had a code for CKD in their EMR. The control group included all individuals without a CKD code (*n* = 1 240 807). Table 1 and Figure 1 show the demographics of people with and without CKD. Males accounted for 36.4% of the CKD group, compared with 49.2% in the control group. People with CKD were older than those without (mean age 74.9 years versus 50.6 years). Prevalence of CKD statistically differed significantly between the youngest and oldest. Using 45–54 years as the reference age group, the adjusted odds ratio (aOR) for CKD presence for those aged 25–34 years was 0.12 (95% CI = 0.10 to 0.15) whereas the aOR for those aged ≥85 years was 45.70 (95% CI = 42.70 to 48.80). People with CKD were more likely to live in the most deprived areas (aOR versus most affluent 1.39, 95% CI = 1.32 to 1.47), with aORs for deprivation and CKD statistically significant from deprivation decile ≥7 (Figure 1).

**Table 1.** Demographics by CKD status, *N* = 1 274 374

**Characteristic**	**CKD (*n*= 33 567)**	**Without CKD (*n*= 1 240 807)**	**OR (95% CI)**	**aOR^a^ (95% CI)**
**Total, *n* (%)**	33 567 (2.6)^b^	1 240 807 (97.4)^b^	–	–

**Sex, male, *n* (%)**	12 225 (36.4)	610 680 (49.2)	0.59 (0.58 to 0.60)	0.81 (0.79 to 0.83)

**Mean age, years (SD)**	74.9 (10.9)	50.6 (16.2)	–	–

**Age group, years, *n* (%)**				
25–34	117 (0.3)	229 477 (18.5)	0.12 (0.10 to 0.15)	0.12 (0.10 to 0.15)
35–44	420 (1.3)	278 929 (22.5)	0.36 (0.32 to 0.40)	0.36 (0.32 to 0.40)
45–54	1054 (3.1)	253 110 (20.4)	1.00	1.00
55–64	3536 (10.5)	216 168 (17.4)	3.93 (3.67 to 4.21)	3.94 (3.68 to 4.22)
65–74	9136 (27.2)	146 356 (11.8)	14.90 (14.10 to 16.00)	14.90 (14.00 to 15.90)
75–84	13 238 (39.4)	86 223 (6.9)	36.90 (34.60 to 39.20)	36.10 (33.90 to 38.50)
≥85	6066 (18.1)	30 544 (2.5)	47.70 (44.60 to 51.00)	45.70 (42.70 to 48.80)

**Deprivation decile, *n* (%)**				
1 (least)	3032 (9.0)	116 316 (9.4)	1.00	1.00
2	3470 (10.3)	123 215 (9.9)	1.08 (1.03 to 1.14)	1.16 (1.10 to 1.22)
3	3197 (9.5)	120 684 (9.7)	1.02 (0.97 to 1.07)	0.94 (0.89 to 0.99)
4	4048 (12.1)	147 848 (11.9)	1.05 (1.00 to 1.10)	1.04 (0.99 to 1.10)
5	3816 (11.4)	142 850 (11.5)	1.02 (0.98 to 1.08)	1.05 (1.00 to 1.10)
6	3213 (9.6)	140 072 (11.3)	0.88 (0.84 to 0.93)	0.93 (0.88 to 0.98)
7	3436 (10.2)	130 990 (10.6)	1.01 (0.96 to 1.06)	1.12 (1.07 to 1.18)
8	3086 (9.2)	104 384 (8.4)	1.13 (1.08 to 1.19)	1.25 (1.18 to 1.32)
9	3453 (10.3)	113 568 (9.2)	1.17 (1.11 to 1.23)	1.26 (1.20 to 1.33)
10 (most)	2816 (8.4)	100 880 (8.1)	1.07 (1.02 to 1.13)	1.39 (1.32 to 1.47)

a *Adjusted for age, sex, and deprivation.*

b *Percentage of entire study population,* N *= 1 274 374.* *aOR = adjusted odds ratio. CKD = chronic kidney disease. OR = odds ratio.*

### CKD and total comorbidity

Markedly higher levels of comorbidity were found in people with CKD compared with controls (98.2% versus 51.8%) in both unadjusted analysis and age-, sex-, and deprivation-adjusted comparisons. Strikingly, only 1.8% of people with CKD had no comorbidities, compared with 48.2% in the control group (Table 2). People with CKD had a higher mean number of comorbidities than people without CKD: 3.8 (standard deviation [SD] 2.2) versus 1.2 (SD 1.6). The mean number of physical comorbidities in people with CKD was 3.4 (SD 1.9) versus 0.9 (SD 1.4) in people without. After age, sex, deprivation adjustment, people with CKD were considerably more likely to have 1 condition (aOR 6.5, 95% CI = 6.0 to 7.1), 2–3 conditions (aOR 15.2, 95% CI = 14.0 to 16.5), 4–6 conditions (aOR 26.6, 95% CI = 24.4 to 28.9), and ≥7 conditions (aOR 41.9, 95% CI = 38.3 to 45.8).

**Table 2.** Number of comorbidities by CKD status, *N* = 1 274 374

**Characteristic**	**CKD (*N*= 33 567)**	**Without CKD (*N*= 1 240 807)**	**Unadjusted OR (95% CI)**	**aOR (95% CI)^a^**
**Mean comorbidities, *n* (SD)**	3.8 (2.2)	1.2 (1.6)	–	–

**Total comorbidities, *n* (%)**				
0	614 (1.8)	598 194 (48.2)	1.00	1.00
1	3553 (10.6)	278 807 (22.5)	12.4 (11.4 to 13.5)	6.5 (6.0 to 7.1)
2–3	12 472 (37.2)	248 971 (20.1)	48.8 (45.0 to 53.0)	15.2 (14.0 to 16.5)
4–6	13 000 (38.7)	99 779 (8.0)	126.9 (117.0 to 137.7)	26.6 (24.4 to 28.9)
≥7	3928 (11.7)	15 056 (1.2)	254.2 (233.1 to 277.2)	41.9 (38.3 to 45.8)

**Mean physical health comorbidities, *n* (SD)**	3.4 (1.9)	0.9 (1.4)	–	–

**Physical comorbidities, *n* (%)**				
0	801 (2.4)	669 088 (53.9)	1.00	1.00
1	4356 (13.0)	275 611 (22.2)	13.2 (12.2 to 14.2)	6.5 (6.0 to 7.0)
2–3	14 130 (42.1)	220 654 (17.8)	53.5 (49.9 to 15.2)	15.2 (14.1 to 16.4)
4–6	12 070 (36.0)	69 280 (5.6)	145.5 (135.4 to 156.4)	28.4 (26.3 to 30.6)
≥7	2210 (6.6)	6174 (0.5)	299.0 (274.7 to 325.4)	49.0 (44.8 to 53.5)

**Mean mental health comorbidities, *n* (SD)**	0.4 (0.8)	0.2 (0.6)		

**Mental comorbidities, *n* (%)**				
0	22 980 (68.5)	1 022 283 (82.4)	1.00	1.00
1	7016 (20.9)	159 527 (12.9)	2.0 (1.9 to 2.0)	1.3 (1.2 to 1.3)
2–3	3464 (10.3)	57 091 (4.6)	2.7 (2.6 to 2.8)	1.4 (1.3 to 1.4)
≥4	107 (0.3)	1906 (0.2)	2.5 (2.05 to 3.04)	1.37 (1.11 to 1.69)

a Adjusted for age, sex, and deprivation. aOR = adjusted odds ratio. CKD = chronic kidney disease. OR = odds ratio.

### CKD and individual concordant and discordant physical comorbidity

**Figure 2.**
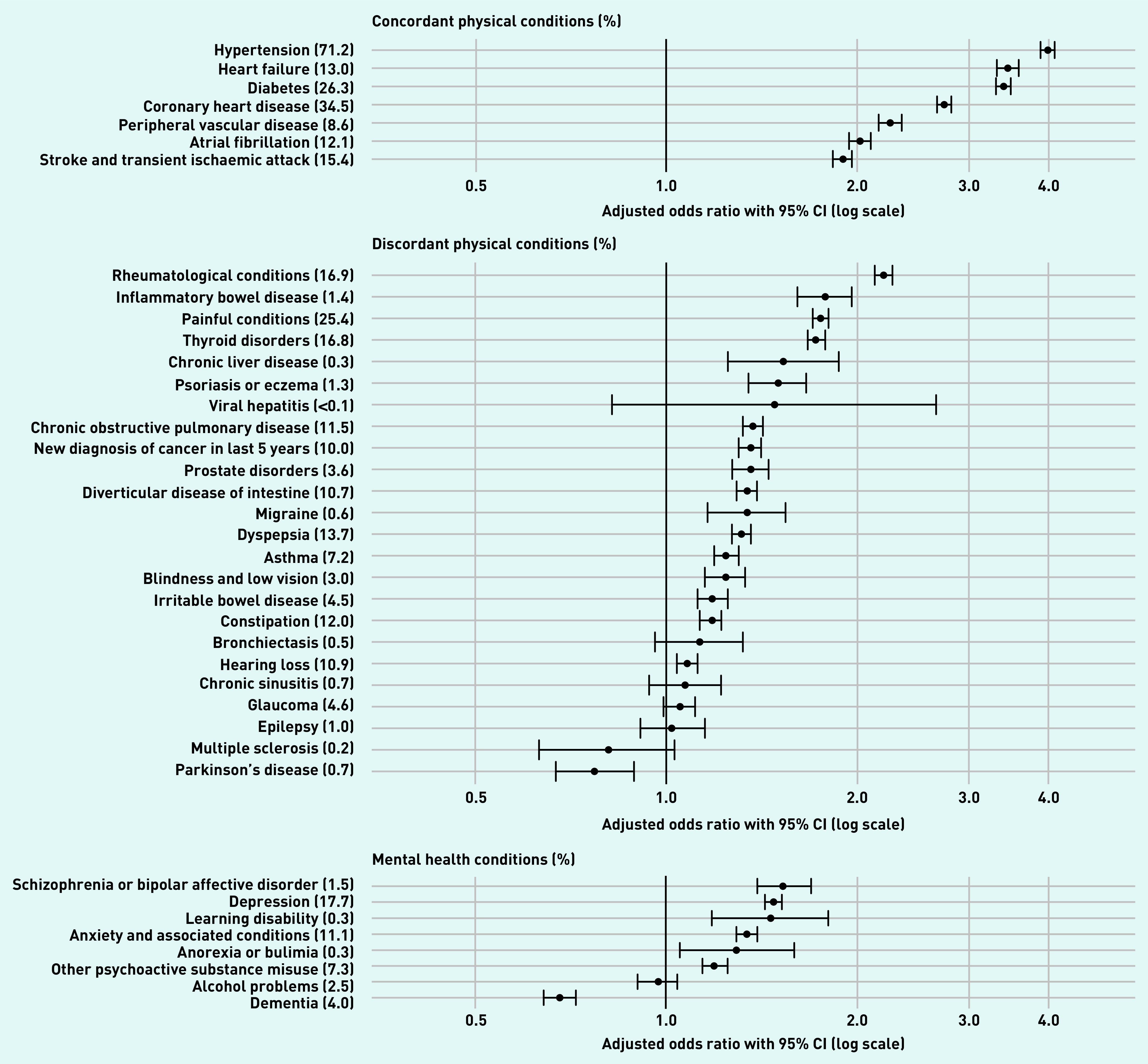
***Age-, sex-, and deprivation-adjusted odds ratios for physical and mental comorbidities.****^a^* *^a^****Disease labels show percentage of people with CKD who also have this disease. CKD = chronic disease.***

Crude prevalence of the most commonly recorded concordant condition, hypertension, was 71.2% (versus 17.0% in controls), followed by CHD with 34.5% (versus 5.6%), and diabetes with 26.3% (versus 5.2%), whereas painful conditions, with a prevalence of 25.4% (versus 9.4%); thyroid disorders 16.8% (versus 5.3%); and dyspepsia 13.7% (versus 6.0%) were the most commonly recorded discordant conditions (Figure 2 and Supplementary Table S1).

All seven concordant conditions and almost all discordant physical health conditions (17 out of 24) were significantly more common in people with CKD after adjustment for age, sex, and deprivation (Figure 2 and Supplementary Table S1). Concordant conditions with the strongest adjusted associations were hypertension (aOR 3.99, 95% CI = 3.89 to 4.09), heart failure (aOR 3.45, 95% CI = 3.32 to 3.59), and diabetes (aOR 3.40, 95% CI = 3.31 to 3.49). Discordant conditions with the strongest associations were rheumatological conditions (aOR 2.20, 95% CI = 2.13 to 2.27), inflammatory bowel disease (IBD) (aOR 1.78, 95% CI = 1.61 to 1.96), and painful conditions (aOR 1.75, 95% CI = 1.70 to 1.80), viral hepatitis, bronchiectasis, chronic sinusitis, glaucoma, epilepsy, and multiple sclerosis had statistically non-significant associations with CKD. The direction of association for Parkinson’s disease (PD) reversed following adjustment (OR 3.33, 95% CI = 2.91 to 3.82 versus aOR 0.77, 95% CI = 0.67 to 0.89).

### CKD and individual mental health comorbidities

Crude prevalence of the most commonly recorded mental health conditions were depression (17.7% versus 10.7% in controls), anxiety and other neurotic stress-related and somatoform disorders (11.1% versus 4.1%), and other psychoactive substance misuse (7.3% versus 3.1%). Of the eight mental health conditions, six had a statistically significant positive association with CKD after adjustment (Figure 2 and Supplementary Table S1). Schizophrenia or bipolar affective disorder (aOR 1.53, 95% CI = 1.39 to 1.69), depression (aOR 1.48, 95% CI = 1.43 to 1.52), and learning disability (aOR 1.46, 95% CI = 1.18 to 1.80) had the strongest associations. Alcohol problems had a statistically non-significant association with CKD. The association between CKD and dementia reversed following adjustment for age, sex, and deprivation, and became negative (OR 4.97, 95% CI = 4.69 to 5.27 versus aOR 0.68, 95% CI = 0.64 to 0.72).

## DISCUSSION

### Summary

The authors of the present analysis of a large, nationally representative primary care dataset found that almost all (98.2%) patients with CKD had comorbidity, including concordant and discordant physical conditions, and mental health conditions. Extreme levels of comorbidity were common in CKD. For example, after adjusting for age, sex, and deprivation, having ≥7 conditions was >40 times more common in the CKD group compared with the rest of the population. Positive associations with CKD were found in all seven concordant physical conditions, 17 of the 24 discordant conditions, and six out of eight mental health conditions after adjustment. Concordant diseases, such as hypertension, heart failure, diabetes, CHD, and PVD, had the highest aORs in the CKD group. This is unsurprising given that hypertension, diabetes, and PVD are known to cause CKD, and CVD processes are accelerated in the context of CKD.^11^ The majority of discordant diseases, with less research evidence base to explain concurrent prevalence in people with CKD, also showed significant associations, with the strongest aORs seen in rheumatological disease, IBD, and pain. Of the mental health conditions, schizophrenia or bipolar affective disorder, depression, and learning disability had the strongest associations with CKD after adjustment.

The present study found that fewer people in the CKD group were male, which could be explained by the higher mean age of the CKD group in the context of lower life expectancy of males compared with females in Scotland.^20^ The study also found that people of lower SES are more likely to have CKD after age and sex adjustment. This is an important finding given that individuals of lower SES have greater mortality owing to comorbid factors that then predict mortality on an independent basis,^21^ including obesity and associated cardiovascular risk factors.^16^

Adjusting for age, sex, and deprivation reversed the direction of association for PD and dementia so both had ORs <1. In the case of PD, a possible explanation might be proposed effects of cigarette smoking,^22^ where those who smoke might be more likely to develop CKD as a result of CVD while being protected from PD. With regard to dementia, this finding could relate to survivor effects given that people without CVD and diabetes comorbidity are more likely to survive to an age where Alzheimer’s disease is very common.

### Strengths and limitations

The study has a number of strengths, notably the use of a large, nationally representative primary care dataset, including almost one-third of the Scottish population. Like all studies using routine data, identification of CKD relied on how well GPs recorded the disease in the EMR and some under-ascertainment is likely. However, CKD was part of the QOF at the time of data extraction, which means GPs were financially incentivised to keep accurate registers. The prevalence of CKD in this study was 2.6% compared with the Global Burden of Disease (GBD) study estimate of 5.2%.^23^ This discrepancy can be explained by the GBD estimate including CKD stages 1 and 2 (accounting for around half of cases) compared with this study examining CKD stages 3 to 5. Data were collected in 2007 and prevalence rates of at least some of the diseases are likely to have changed given the 13-year interim period, though patterns of disease relationships are likely to be similar. Clinical detail, such as the stratification of CKD severity by estimated glomerular filtration rate and proteinuria, were not available. However, the present study included of a wide range of concordant and discordant physical and mental health conditions, allowing an in-depth analysis of the existence of comorbidities by number and type. Because of the cross-sectional nature of the dataset, causal pathways between variables could not be identified. Comparison made with number of comorbidities between those with CKD and those without may not be a direct comparison because having any one condition already may increase the risk of having other comorbidities. However, in a comparison between those with CKD and those with any other condition it would be difficult to apply any statistical test because both groups are overlapping populations and the authors recognise this as a limitation, but it remains clear that people with CKD have very high levels of comorbidity.

### Comparison with existing literature

A small number of studies have measured the burden of comorbidity in CKD; however, to the best of the authors’ knowledge, no existing studies have included a control population. Fraser *et al* examined comorbidity in a smaller population of 1741 people with CKD stage 3, and looked at the prevalence of a smaller number of comorbidities,^11^ which were not divided into concordant or discordant relationship with CKD.^5^ The study reported that comorbidity was seen in 96% of those with CKD, and 40% of those with CKD had ≥2 comorbidities.

Several studies have examined the relationship between CKD and concordant conditions including CVD and diabetes.^24^^–^^27^ Few have examined associations with discordant physical and mental health conditions that are addressed in detail in the present study. Bowling *et al* studied the association between number of chronic conditions stratified by the presence of ≥1 discordant conditions.^4^ They found that at least one discordant comorbidity was associated with higher risks of emergency department visits and hospitalisation. A large Canadian study by Tonelli *et al* studied half a million people with CKD and found that adverse clinical outcomes including mortality, hospitalisation, and myocardial infarction were common in all types of comorbidity including concordant, discordant, and mental health conditions.^12^ A recent systematic review and meta-analysis of multimorbidity and adverse clinical outcomes concluded that there is an association between multimorbidity and increased mortality and morbidity in people with CKD.^6^ A key recommendation of the systematic review for future research was describing the prevalence of a wide range of comorbidities associated with CKD, which is addressed in the present study.

### Implications for research and practice

High levels of comorbidity are commonplace in people with CKD, which means that complex and discordant treatment regimens are likely to be common. These regimens can be challenging to adhere to in the context of poor coping mechanisms, and a lack of knowledge about self-management strategies and social support.^28^ Clinicians can also find the management of CKD in the context of multimorbidity challenging, given that the majority of clinical guidance relates to single-disease models, often with conflicting advice between conditions. Such guidance can drive cumulative polypharmacy, without providing direction on how best to prioritise recommendations for individuals in whom treatment burden will sometimes be overwhelming.^29^

It is clear that integrated guidance and combined generalist and specialist expertise are required to ensure that appropriate care is delivered to people with CKD, recognising the complexity of their health needs. Further prospective cohort studies are required to determine the sequence in which different types of comorbidities and individual conditions emerge over the life course in people with CKD and evaluate generalist approaches to CKD care.
